# Prostate external beam radiotherapy combined with high-dose-rate brachytherapy: dose-volume parameters from deformably-registered plans correlate with late gastrointestinal complications

**DOI:** 10.1186/s13014-016-0719-2

**Published:** 2016-10-31

**Authors:** Calyn R. Moulton, Michael J. House, Victoria Lye, Colin I. Tang, Michele Krawiec, David J. Joseph, James W. Denham, Martin A. Ebert

**Affiliations:** 1 School of Physics (M013), University of Western Australia, 35 Stirling Highway, Crawley, WA 6009 Australia; 2 Radiation Oncology, Sir Charles Gairdner Hospital, Hospital Avenue, Nedlands, WA 6009 Australia; 3 School of Surgery, University of Western Australia, 35 Stirling Highway, Crawley, WA 6009 Australia; 4 School of Medicine and Population Health, University of Newcastle, University Drive, Callaghan, NSW 2308 Australia

**Keywords:** Deformable registration, Gastrointestinal toxicity, Distribution-adding

## Abstract

**Background:**

Derivation of dose-volume correlated with toxicity for multi-modal treatments can be difficult due to the perceived need for voxel-by-voxel dose accumulation. With data available for a single-institution cohort with long follow-up, an investigation was undertaken into rectal dose-volume effects for gastrointestinal toxicities after deformably-registering each phase of a combined external beam radiotherapy (EBRT)/high-dose-rate (HDR) brachytherapy prostate treatment.

**Methods:**

One hundred and eighteen patients received EBRT in 23 fractions of 2 Gy and HDR (TG43 algorithm) in 3 fractions of 6.5 Gy. Results for the Late Effects of Normal Tissues — Subjective, Objective, Management and Analytic toxicity assessments were available with a median follow-up of 72 months. The HDR CT was deformably-registered to the EBRT CT. Doses were corrected for dose fractionation. Rectum dose-volume histogram (DVH) parameters were calculated in two ways. (1) Distribution-adding: parameters were calculated after the EBRT dose distribution was 3D-summed with the registered HDR dose distribution. (2) Parameter-adding: the EBRT DVH parameters were added to HDR DVH parameters. Logistic regressions and Mann-Whitney U-tests were used to correlate parameters with late peak toxicity (dichotomised at grade 1 or 2).

**Results:**

The 48–80, 40–63 and 49–55 Gy dose regions from distribution-adding were significantly correlated with rectal bleeding, urgency/tenesmus and stool frequency respectively. Additionally, urgency/tenesmus and anorectal pain were associated with the 25–26 Gy and 44–48 Gy dose regions from distribution-adding respectively. Parameter-adding also indicated the low-mid dose region was significantly correlated with stool frequency and proctitis.

**Conclusions:**

This study confirms significant dose-histogram effects for gastrointestinal toxicities after including deformable registration to combine phases of EBRT/HDR prostate cancer treatment. The findings from distribution-adding were in most cases consistent with those from parameter-adding. The mid-high dose range and near maximum doses were important for rectal bleeding. The distribution-adding mid-high dose range was also important for stool frequency and urgency/tenesmus. We encourage additional studies in a variety of institutions using a variety of dose accumulation methods with appropriate inter-fraction motion management.

**Trial registration:**

NCT NCT00193856. Retrospectively registered 12 September 2005.

**Electronic supplementary material:**

The online version of this article (doi:10.1186/s13014-016-0719-2) contains supplementary material, which is available to authorized users.

## Background

External beam radiotherapy (EBRT) with a high-dose-rate brachytherapy (HDR) boost dose is used to treat prostate cancer patients [1]. This treatment and other radiotherapy treatments are planned with consideration of the dose-volume parameters and subsequent constraints associated with acceptable levels of normal tissue toxicity [2]. However, typically the phases of combined EBRT/HDR are planned separately [3]. Constraints on the total planned dose from the two phases would be appropriate for reducing normal tissue toxicity [4]. Constraints could be applied for each phase; however, this is susceptible to anatomical differences between the planning CTs.

When adjustments for anatomical changes are not included, the relevance of plans based on dose-volume constraints depends on how well the planned dose reflects the delivered dose [5]. Hence, studies in other radiotherapy contexts have incorporated dose accumulation [6, 7]. Simple crude addition of the separately planned doses from two modalities is not valid as the anatomy in the CT image study sets may be misaligned due to variations in reference coordinate systems, displacements, deformations and shrinkage [8]. Consequently, a rigid registration is used to align the reference coordinate systems and then deformable image registration (DIR) is applied to adjust for deformations and shrinkage [9, 10]. Additionally, the doses for different fraction schedules should be converted to the equieffective dose given in a reference X Gy per fraction (EQDX_α/β_ Gy) as this adjusts for the biologically non-equivalent fractionation schedules [5, 11, 12].

Adjusting for anatomical differences between planning CTs and subsequently accumulating the phases of planned dose more accurately may allow dose-volume parameters to be more appropriately correlated with toxicity [2, 13]. Studies accumulating the rectum dose from phases of a combined EBRT/HDR prostate treatment by applying deformable registration are lacking. This study uses data from combined EBRT/HDR prostate cancer treatments, which were subject to multicentre trial guidelines, to assess whether the rectum dose-histogram parameters extracted after applying deformable registration are correlated with late gastrointestinal toxicities.

## Methods

### Patient data

This study included 118 prostate cancer patients (tumour T stage ≥ 2a) who were treated with EBRT followed by HDR at Sir Charles Gairdner Hospital in the period 2004 to 2008. These patients were treated as part of the Trans-Tasman Radiation Oncology Group (TROG) 03.04 Randomized Androgen Deprivation and Radiotherapy (RADAR) trial [14, 15]. The patient criteria and treatment methodology for the RADAR trial have previously been detailed [14, 15]. Aspects of the combined EBRT/HDR treatment process have previously been described [16]. The four-field EBRT plans for a prescription dose of 46 Gy in 23 daily fractions were created in the Elekta XiO treatment planning system (Elekta AB, Stockholm, Sweden). The HDR plans for a prescription dose of 19.5 Gy in 3 fractions across 2 days were created in the BrachyVision treatment planning system (Varian Medical Systems, Palo Alto, US) using the TG43 formalism [17]. Additional file 1 (Supplement A) provides additional patient and treatment details.

The external wall of the rectum was manually delineated by treating-clinicians in the HDR CTs using BrachyVision and in the EBRT CTs using the Elekta Focal contouring system (Elekta AB, Stockholm, Sweden). Author with initials MK reviewed rectum outlines for consistency between patients. For rectum contouring, the inferior-superior limits of the rectum were the rectosigmoid flexure and the last slice where the ischial tuberosities were visible. Patients did not commonly require bowel preparation. Examples of the planning CTs and structures for EBRT and HDR TG43 physical dose plans are provided in Additional file 1 (Supplement B, Figures A1 and A2).

### Toxicity outcomes

Patients were assessed for various gastrointestinal toxicities at baseline (randomisation) and subsequent time points after randomisation. The median of the most recent patient follow-ups was 72 months (range 12–96 months). The Late Effects of Normal Tissue — Subjective, Objective, Management, and Analytic (LENT SOMA) scales were used to assess rectal bleeding, urgency and tenesmus, stool frequency, diarrhoea, anorectal pain and completeness of evacuation [18]. Proctitis was scored by clinicians according to the Common Toxicity Criteria for Adverse Events (CTCAE version 2) [19]. Additional file 1 (Supplement C, Table A2) provides a summary of the grading systems.

**Fig. 1:**
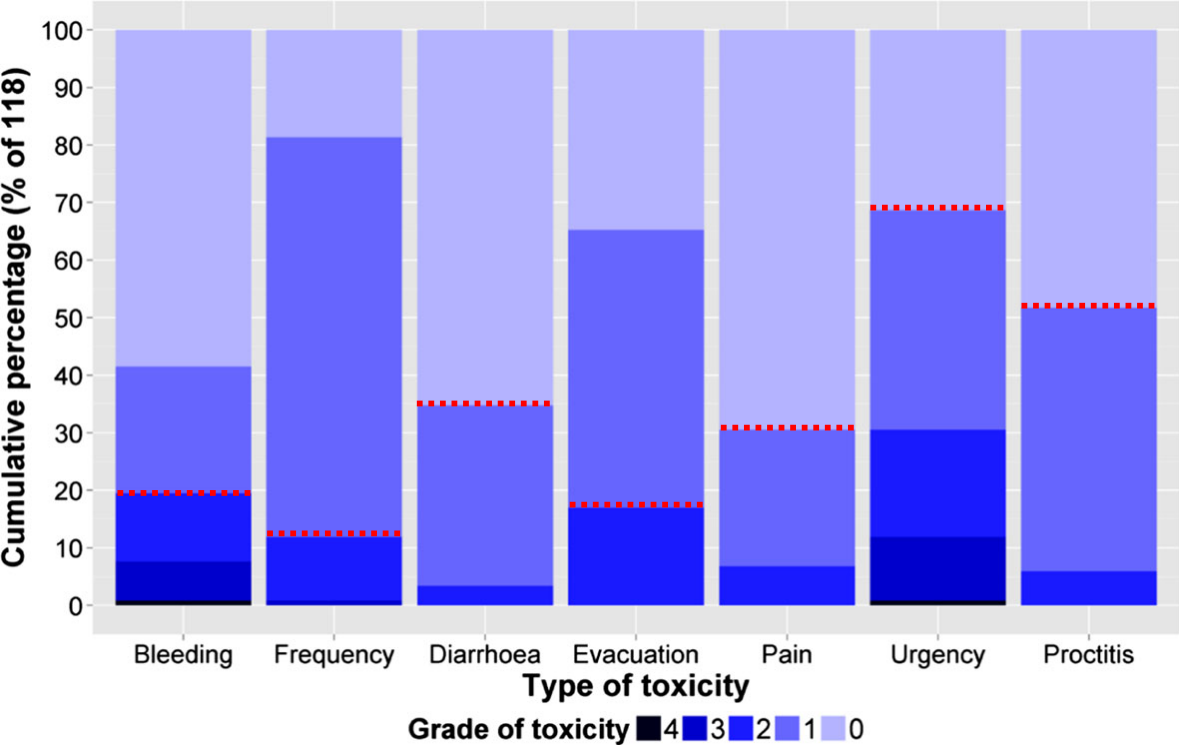
Late peak toxicity grades for various toxicity types over the follow-up period. The toxicity types (abbreviation) are rectal bleeding (bleeding), CTC proctitis (proctitis), stool frequency (frequency), diarrhoea, urgency/tenesmus (urgency), anorectal pain (pain) and completeness of evacuation (evacuation). The toxicity rates are reported as cumulative percentages of the 118 patients. The thresholds for subsequently grouping patients into toxicity/no toxicity groups are indicated by the red dashed lines

Late peak toxicity was calculated for the period from 3 months after radiation therapy and onwards. Figure 1 provides a summary of the late peak toxicity event rates for the follow-up period. Patients were classified to a toxicity group if the late peak toxicity was at least a certain grade (threshold for dichotomisation). In the interest of modelling a moderate severity of toxicity the threshold was grade 2 for rectal bleeding, stool frequency and completeness of evacuation. The threshold was grade 1 for diarrhoea, anorectal pain, proctitis, urgency and tenesmus due to low toxicity rates for grade ≥ 2 toxicity and/or a lack of significance for grade ≥ 2 toxicity. Alternatively, the chosen thresholds for toxicities are indicated in Fig. 1. The analysis was repeated using the prevalence of toxicity at 36 months post-randomisation. This did not reveal any additional trends for dose-histogram effects and so is reported no further.

### Registration process

**Fig. 2:**
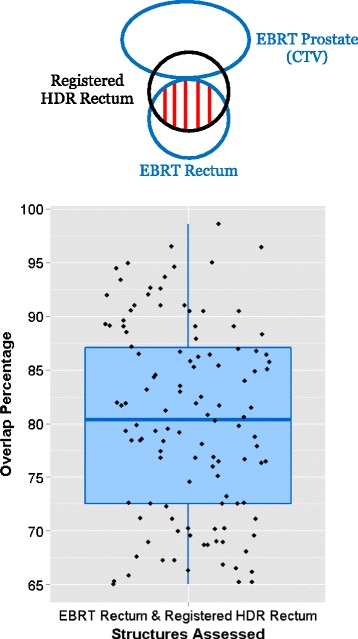
Registration evaluation using structure overlaps. Illustration of the structure overlap metric used to assess major misalignment of rectum volume (*Top*). Overlap of the EBRT rectum/registered HDR rectum was expressed as a percentage of the volume of the registered HDR rectum. Structure overlap results for the 118 patients after the rigid plus multi-pass DIR are provided (*Bottom*)

The HDR CT was registered to the EBRT CT using rigid registration followed by a B-splines multi-pass DIR in Velocity Advanced Imaging 2.8.1 (Varian Medical Systems, Palo Alto, US) [20]. The registration process has been described in detail previously [20, 21]. Visual inspections for the 118 patients were undertaken by authors (initials CRM, VL and CIT) and did not identify any major registration misalignments (e.g. Additional file 1 [Supplement D, Figure A3]). The registrations were quantitatively evaluated for each patient in this study using the overlap of the EBRT rectum and registered HDR rectum (expressed as a percentage of the volume of the registered HDR rectum). As illustrated in Fig. 2, the median overlap is 80.4 % for alignment of EBRT/registered HDR rectum structure volumes. A general structure overlap of 70 % is considered to be the starting point for satisfactory structure-correspondence in the radiotherapy context [22, 23]. The registrations have also previously been extensively evaluated using structure-correspondence metrics, image similarity metrics and qualitative visual inspection by authors (initials CRM, VL and CIT) [21].

### Obtaining dose-volume histograms

The EBRT and registered HDR 3D-doses were imported into MATLAB™ R2010a (The MathWorks Inc., Massachusetts, US) and the Computational Environment for Radiotherapy Research (CERR, version 4.1) [24]. The voxel doses were converted to equieffective doses given in a reference 2 Gy per fraction using the linear-quadratic model [11] with an alpha-beta ratio of 3 Gy for the rectum [2]. The analysis was also performed for an alpha-beta ratio of 5.4 Gy to check the sensitivity of results to the upper limit published for the rectum [25]. The EBRT dose was summed voxel-by-voxel with the registered HDR dose (i.e. accumulated). The rectum dose-volume histograms (DVH) in 1 Gy bins from 1 to 80 Gy were extracted for the total registered dose (with EBRT rectum structure), the unregistered EBRT dose (with EBRT rectum structure) and the unregistered HDR dose (with the HDR rectum structure). The parameters extracted from the rectum DVHs were the V_X_ (percentage of the rectal volume receiving at least X Gy after applying an alpha-beta ratio) and D_X%_ (minimum dose to the most irradiated X percent of the rectal volume after applying an alpha-beta ratio). The V_X_ and D_X%_ were calculated using the total registered dose and the EBRT rectum structure (‘distribution-adding’). Additionally, the D_X%_ was alternatively calculated by adding the EBRT D_X%_ to the unregistered HDR D_X%_ using the corresponding rectum structures (‘parameter-adding’).

### Response modelling

For each type of toxicity, univariate logistic regression was applied at each V_X_ to obtain an odds ratio (OR) for the increase in toxicity probability per 5 % absolute increase in volume [26]. 95 % confidence intervals (CI) for odds ratios were calculated using bootstrapping with 10,000 resamples from the toxicity and no toxicity groups. Odds ratios were considered significant if the 95 % CIs did not include the value of one. Mann-Whitney U-tests were used to determine whether the median V_X_ (or D_X%_) values for the toxicity and no toxicity groups were significantly different (*p*-value < 0.05). This analysis was performed in MATLAB™ R2010a (The MathWorks Inc., Massachusetts, US).

Clinical risk factors were not included in dose-response modelling as a previously published analysis determined that clinical covariates did not significantly influence late toxicities for patients in the RADAR trial [27]. An equivalent analysis for the 118 patients in this study confirmed that clinical covariates did not significantly influence late toxicities. The clinical factors considered were age, tumour T stage, Gleason score, initial PSA, risk group, number of HDR catheters, colorectal disorders, hypertension, diabetes, smoking, use of statins, ACE inhibitors and anti-coagulants.

## Results

Unless it is stated otherwise, all figures and tables in this section report distribution-adding dose values in a reference 2 Gy per fraction using an α/β of 3 Gy.

**Fig. 3:**
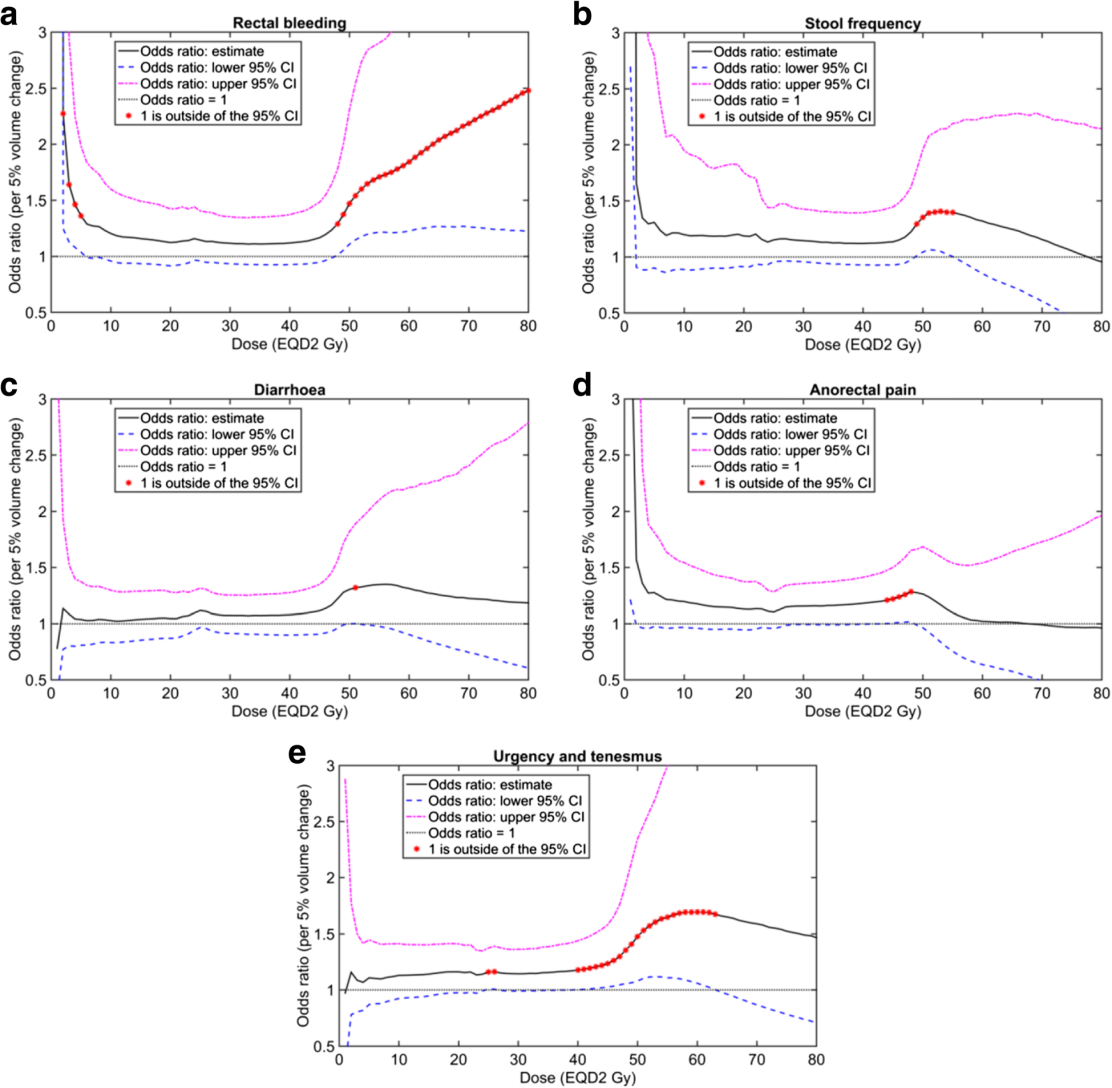
Odds ratios from univariate ordinal regression of distribution-adding V_X_ and peak late toxicity. The toxicities are rectal bleeding (**a**), stool frequency (**b**), diarrhoea (**c**), anorectal pain (**d**) and urgency/tenesmus (**e**). The peak late toxicities for rectal bleeding and stool frequency were dichotomised at grade 2 whereas diarrhoea, anorectal pain and urgency/tenesmus were dichotomised at grade 1. A red dot is used to indicate the doses at which odds ratios are significantly different from a value of one (95 % confidence intervals do not include one). *Abbreviations*: V_X_, percentage of the rectal volume receiving at least X Gy after applying an α/β = 3 Gy; EQD2 Gy, equivalent dose in 2-Gy fractions using α/β = 3 Gy; 95 % CI, 95 % confidence interval

Figure 3 provides the logistic regression odds ratio results for late rectal bleeding, stool frequency, diarrhoea, anorectal pain, urgency and tenesmus respectively. Figure 3 includes an indication for distribution-adding dose levels where the 95 % confidence intervals for the odds ratio did not include a value of one. For completeness of evacuation and proctitis, the odds ratios are not significantly different from one at any dose levels (Additional file 1 [Supplement E, Figure A4]).

**Fig. 4:**
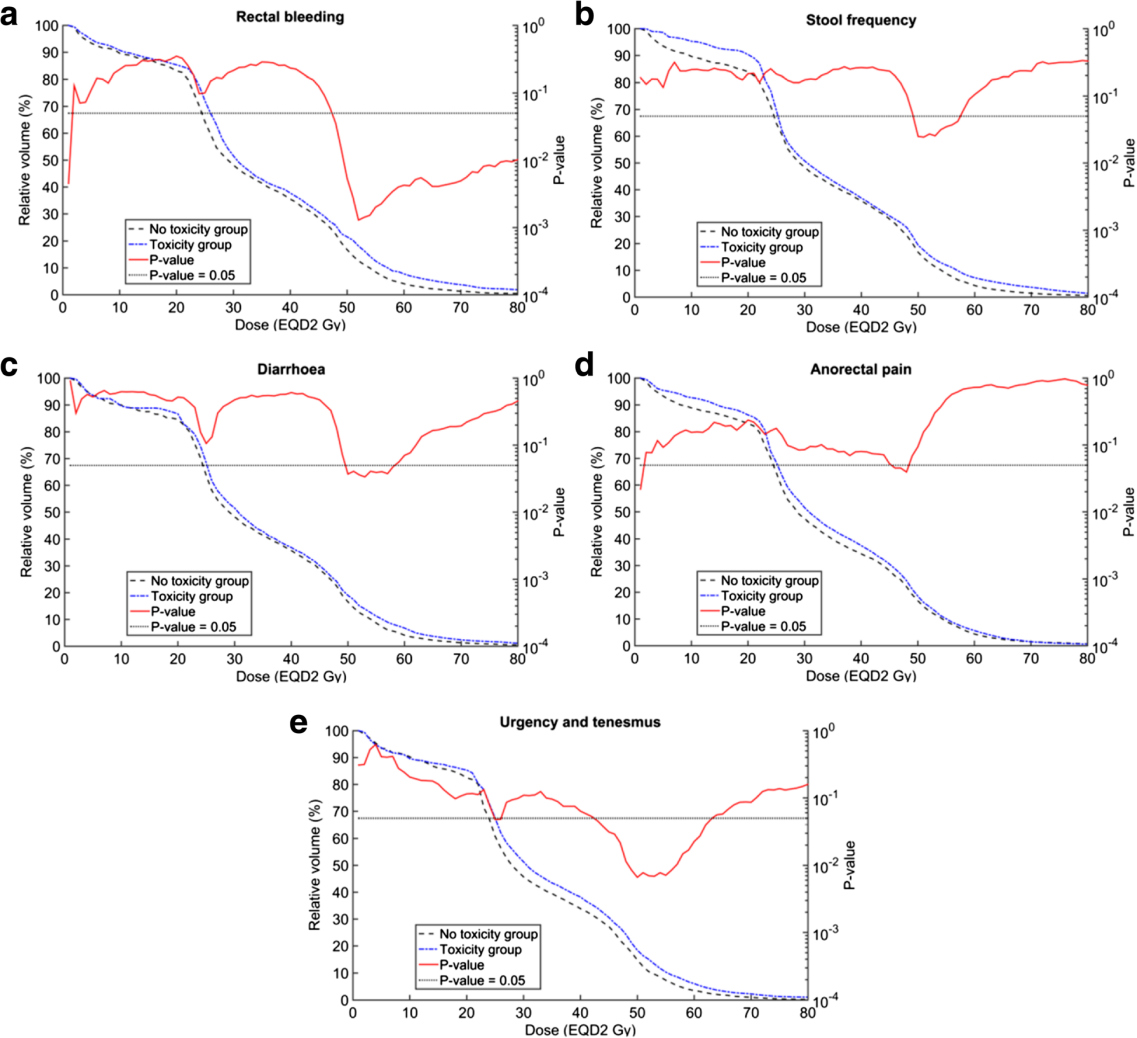
Median distribution-adding V_X_ for the toxicity and no toxicity groups. The toxicity groups are based on peak late toxicity. The peak late toxicities for rectal bleeding (**a**) and stool frequency (**b**) were dichotomised at grade 2 whereas diarrhoea (**c**), anorectal pain (**d**) and urgency/tenesmus (**e**) were dichotomised at grade 1. The red curve and *p*-value axis indicate doses at which median V_X_ values for the toxicity and no toxicity groups are significantly different (*p*-value < 0.05). *Abbreviations*: V_X_, percentage of the rectal volume receiving at least X Gy after applying an α/β = 3 Gy; EQD2 Gy, equivalent dose in 2-Gy fractions using α/β = 3 Gy

**Fig. 5:**
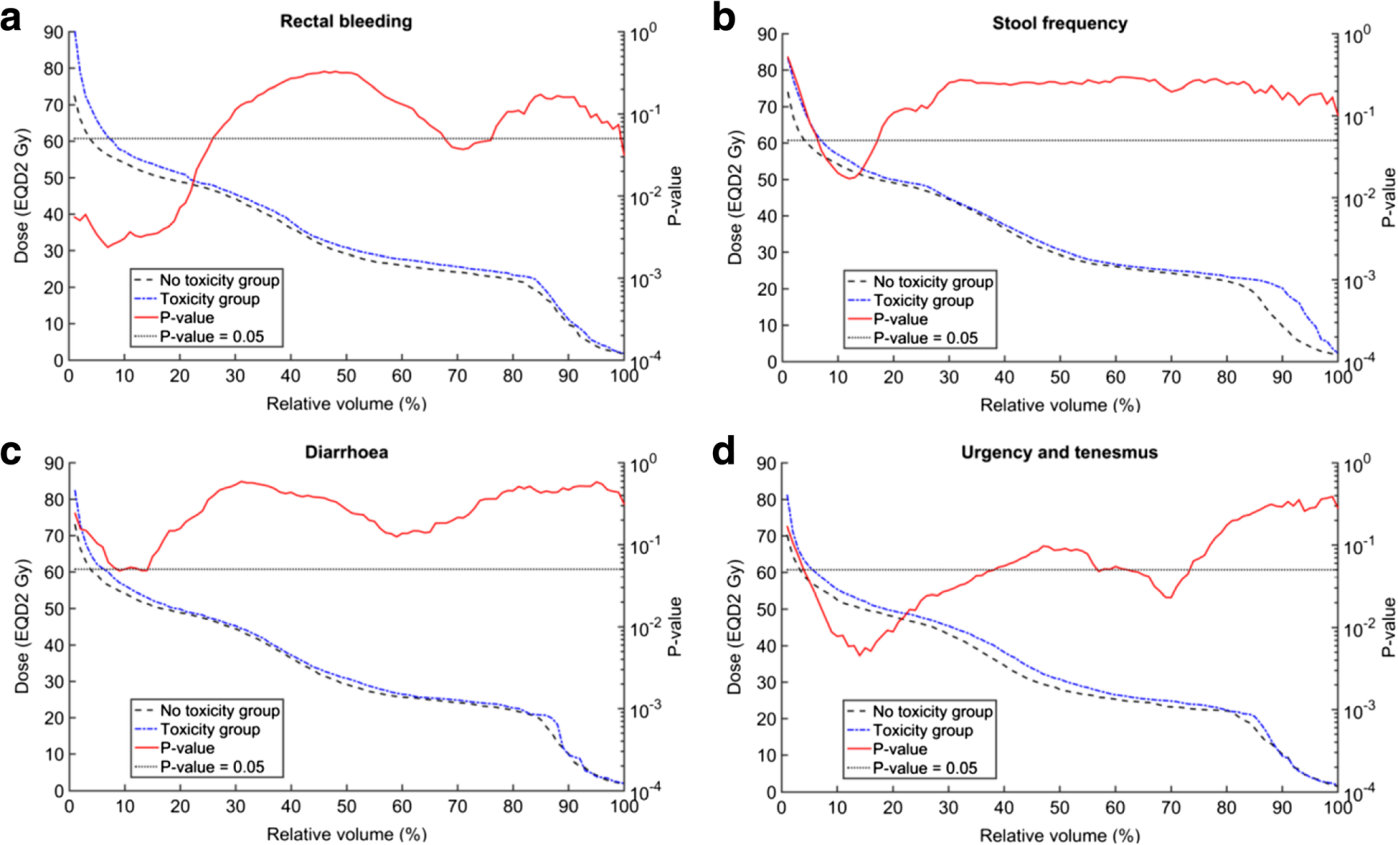
Median distribution-adding D_X%_ for the toxicity and no toxicity groups. The peak late toxicities for rectal bleeding (**a**) and stool frequency (**b**) were dichotomised at grade 2 whereas diarrhoea (**c**) and urgency/tenesmus (**d**) were dichotomised at grade 1. The red curve and *p*-value axis indicate doses at which median D_X%_ values for the toxicity and no toxicity groups are significantly different (*p*-value < 0.05). *Abbreviations*: D_X%_, minimum dose to the most irradiated X percentage of rectal volume after applying an α/β = 3 Gy; EQD2 Gy, equivalent dose in 2-Gy fractions using α/β = 3 Gy

Figure 4 provides the distribution-adding V_X_ results for late rectal bleeding, stool frequency, diarrhoea, anorectal pain, urgency and tenesmus. Figure 5 provides the corresponding distribution-adding D_X%_ results for late rectal bleeding, stool frequency, diarrhoea, urgency and tenesmus. Figures 4 and 5 include an indication of dose and volume levels for which there was a significant difference between the toxicity and no toxicity groups (*p*-value < 0.05). For completeness of evacuation and proctitis, there are no significant differences between the toxicity and no toxicity group results at any distribution-adding V_X_ (Additional file 1 [Supplement E, Figure A5]). For completeness of evacuation, anorectal pain and proctitis, there are no significant differences between the toxicity and no toxicity group results at any distribution-adding D_X%_ (Additional file 1 [Supplement E, Figure A6]).

Table 1 summarises the important distribution-adding doses for the odds ratios and the important distribution-adding doses (volumes) for the V_X_ (D_X%_) results. Additionally, Table 1 summarises the important volumes for the D_X%_ obtained by parameter-adding (alternatively, see Additional file 1 [Supplement F, Figure A7] for full results for the D_X%_ obtained by parameter-adding). The D_8-13%_ and D_5-38%_ become important for diarrhoea and urgency/tenesmus respectively when distribution-adding is used instead of parameter-adding. The parameter-adding D_59-70%_ and D_58-73%_, which were significant for proctitis and stool frequency respectively, are not significant when distribution-adding is used. However, the D_56-74%_ becomes important for urgency/tenesmus when distribution-adding is used instead of parameter-adding. Similar trends for significance of D_X%_ are found for all other toxicities regardless of whether distribution-adding or parameter-adding is used.

**Table 1:** Summary of which parameters (odds ratios, V_X_ and D_X%_) correlate with toxicity

Toxicity type	Distribution-adding			Parameter-adding
	Odds ratio^a^	V_X_^b^	D_X%_^c^	D_X%_^c^
Rectal bleeding	01–05 Gy 48–80 Gy	48–80 Gy	01–25 % 68–78 %	01–25 % 62–78 %
Stool frequency	49–55 Gy	49–57 Gy	07–18 %	02–24 % 58–73 %
Diarrhoea	51 Gy	50–59 Gy	08–13 %	None
Completeness of evacuation	None	None	None	None
Anorectal pain	44–48 Gy	45–48 Gy	None	None
Urgency/tenesmus	25–26 Gy 40–63 Gy	25–27 Gy 43–64 Gy	05–38 % 56–74 %	None
Proctitis	None	None	None	59–70 %

*Abbreviations*: *V*_*X﻿*_ percentage of the rectal volume receiving at least X EQD2 Gy after applying an α/β = 3 Gy, *D*_*X*%_ minimum EQD2 Gy dose to the most irradiated X percentage of rectal volume after applying an α/β = 3 Gy, *EQD2 Gy* equivalent dose in 2-Gy fractions using α/β = 3 Gy, *α*/*β* alpha-beta ratio

^a^Doses at which odds ratios were significantly different from a value of one

^b^Doses at which the median V_X_ for toxicity and no toxicity groups were significantly different

^c^Volumes at which the median D_X%_ for toxicity and no toxicity groups were significantly different

Table 2 summarises the influence of using an α/β of 5.4 Gy instead of 3 Gy. Alternatively, Additional file 1 (Supplement G, Figures A8, A9 and A10) provide the odds ratio, V_X_ and D_X%_ results when distribution-adding and an α/β of 5.4 Gy are used. Also, Additional file 1 (Supplement H, Figure A11) alternatively provides the D_X%_ results obtained by parameter-adding with an α/β of 5.4 Gy. The odds ratio at EQD2_α/β_ 51 Gy for diarrhoea is no longer significant if an α/β of 5.4 Gy is used instead of 3 Gy. However, similar trends for significance of odds ratios, V_X_ and D_X%_ are found for all other toxicities regardless of whether the α/β is 3 or 5.4 Gy.

**Table 2:** Influence of the applied alpha-beta ratio on the findings for odds ratios, V_X_ and D_X%_

Toxicity type	Findings for α/β = 5.4 Gy compared to α/β = 3 Gy^a^
Rectal bleeding	Similar trends
Stool frequency	Similar trends
Diarrhoea	Odds ratios not significant for α/β = 5.4 Gy
Completeness of evacuation	No significance for α/β = 3 Gy or α/β = 5.4 Gy
Anorectal pain	Similar trends
Urgency/tenesmus	Similar trends
Proctitis	Similar trends

*Abbreviations*: *V*_*X*_ percentage of the rectal volume receiving at least X EQD2 Gy after applying an α/β = 3 or 5.4 Gy, *D*_*X*%_ minimum EQD2 Gy dose to the most irradiated X percentage of rectal volume after applying an α/β = 3 or 5.4 Gy, *EQD2 Gy* equivalent dose in 2-Gy fractions using α/β = 3 or 5.4 Gy, *α*/*β* alpha-beta ratio

^a^Indication of whether significance still exists when an α/β of 5.4 Gy is used instead of 3 Gy

## Discussion

### It is important to explore dose-toxicity modelling in a variety of registration contexts

This study is the first to use registration-based distribution-addition to obtain accumulated rectum dose-histogram parameters for combined EBRT/HDR prostate cancer treatment and to then correlate the resulting parameters with gastrointestinal toxicities. Studies have estimated the accumulated rectum dose for combined EBRT/HDR prostate cancer treatment without applying deformable registration [3, 7]. However, Kikuchi et al. [12] acknowledged that deformable image registration should be part of a more accurate method of accumulating the rectum dose. This current study improved upon these studies by applying deformable image registration and then correlating accumulated rectum dose with various gastrointestinal toxicities. This study acknowledges the uncertainties of deformable image registration. Subsequently, it compares the findings for distribution-adding with the findings for parameter-adding. Given the potential uncertainties of deformable registration it is important for registration-based dose-toxicity modelling to be published for a variety of studies. This would allow a multi-institutional comparison of findings to include the confounding factors associated with different registration algorithms, registration circumstances, associated inter-fraction motion constraints and diversity in treatment techniques.

### Studies had identified important dose-volume metrics for a variety of prostate radiotherapy techniques

The volume receiving certain doses and the magnitude of dose delivered to volumes have been associated with late gastrointestinal toxicities, typically rectal bleeding, scored after a number of prostate radiotherapy techniques including EBRT only, HDR only, low-dose-rate brachytherapy (LDR) only, combined EBRT/HDR and combined EBRT/LDR [2–4, 26, 28–45]. Table 3 summarises the important dose-response findings for the previously mentioned studies and the findings for this study.

**Table 3:** Summary of findings from previous studies and the current study for various late gastrointestinal toxicities

Toxicity	Dose-volume consideration	Reference	RT technique
Rectal toxicity	Constrain the V_30-70 Gy_	[28, 34–36]	HDR, EBRT
	Constrain the V_40 Gy_ and V_65-80 Gy_	[37]	EBRT
	Constrain the V_100%_	[38]	HDR
	Limit the D_1cc-10cc_	[34]	HDR
	D_5%-90%_ were not significant	[34]	HDR
Rectal bleeding	*Limit the high*/*near maximum doses*	*Current study*	*EBRT*+*HDR*
	*Limit doses* > *48 Gy*	*Current study*	*EBRT*+*HDR*
	*Some association with low doses* (*0*–*5 Gy*)	*Current study*	*EBRT*+*HDR*
	Constrain the V_40-80 Gy_	[2, 26, 28–31, 37, 39–41]	EBRT
	Limit the D_2cc_ and near maximum doses	[4, 42, 43]	EBRT+HDR, LDR, EBRT+LDR
	Near maximum doses were not significant	[3]	EBRT+HDR
	Limit doses > 30 Gy	[28, 30, 32, 33]	EBRT
	Constrain the V_30%_, V_50%_, V_80%_ and V_90%_	[44]	EBRT
	Constrain the V_10%_, V_30%_ and V_50%_	[45]	EBRT+HDR
Stool frequency	*Limit the mid*-*high dose range* (*49*–*57 Gy*)	*Current study*	*EBRT*+*HDR*
	Limit the low-mid dose range (4–38 Gy)	[30]	EBRT
	Constrain the V_50-60 Gy_	[26]	EBRT
	Constrain the V_40 Gy_	[32]	EBRT
Diarrhoea	*Some association with mid*-*high doses* (*50*–*59 Gy*)	*Current study*	*EBRT*+*HDR*
	Limit the low-mid doses (22–32 Gy)	[30]	EBRT
Completeness of evacuation	*No dose range is significant*	*Current study*	*EBRT*+*HDR*
	Limit the low-mid doses (12–36 Gy)	[30]	EBRT
Anorectal pain	*Some association with mid*-*dose range* (*45*–*48 Gy*)	*Current study*	*EBRT*+*HDR*
Urgency/tenesmus	*Limit the mid*-*high doses* (*43*–*64 Gy*)	*Current study*	*EBRT*+*HDR*
	*Some association with low doses* (*25*–*27 Gy*)	*Current study*	*EBRT*+*HDR*
	Constrain the V_40-60 Gy_	[26]	EBRT
	Constrain the V_25-75 Gy_	[37]	EBRT
	Limit the low-mid doses (5–38 Gy)	[30]	EBRT
Proctitis	*No dose range is significant*	*Current study*	*EBRT*+*HDR*
	Constrain the V_40-70 Gy_	[26]	EBRT

*Abbreviations*: *RT* radiotherapy, *EBRT* external beam radiotherapy, *HDR* high-dose-rate brachytherapy, *LDR* low-dose-rate brachytherapy, *V*_*X Gy*_ percentage of the rectal volume receiving at least X Gy, *V*_*X*%_ percentage of the rectal volume receiving at least X% of the prescription dose, *D*_*X*%_ minimum dose to the most irradiated X% of rectal volume, *D*_*Xcc*_ minimum dose to the most irradiated X cubic centimetres of rectal volume, *cc* cubic centimetres

The previously mentioned studies commonly suggested that the incidence of late rectal bleeding following prostate radiotherapy can be reduced by constraining the volume of the rectum receiving high doses (e.g. [4, 28, 42, 43, 46]). Additionally, some of the studies have correlated the mid and low-mid dose regions with late rectal bleeding [28, 30, 32, 33] and stool frequency/urgency/tenesmus [26, 30, 32, 37] respectively. Consequently, the mid-high rectum doses in prostate EBRT are typically managed through constraints on the V_40-75 Gy_ [28, 46] whereas treatments involving prostate brachytherapy (HDR or LDR) should consider the high rectum doses via the V_70%-100%_, D_1cc_, D_2cc_ and/or near maximum dose [4, 47–49] due to high-dose hot spots associated with radioactive sources. For treatments involving prostate HDR, the importance of low-dose regions has been explained in terms of considerable inter-patient variation in rectal gas and the distance from the prostate to the anterior rectal wall [45]. The instances where the V_80 Gy_ and V_90 Gy_ have been identified as important for prostate cancer treatments involving EBRT only were related to homogenous irradiation of volumes with hypofractionated doses [45]. The sections to follow will discuss the findings of this study, summarised in Table 3, relative to findings of the previously mentioned dose-response studies, which are also summarised in Table 3.

### The findings indicate a serial response for rectal bleeding

In agreement with other studies [2, 28–30] the high-dose metrics for the rectum were significantly correlated with rectal bleeding for both distribution-adding and parameter-adding. The significant correlation between near maximum dose metrics for the rectum and rectal bleeding indicates the dose-volume effects follow a serial response. The confirmation of the expected importance of near maximum doses after registration is important as a previous study without registration did not find any significance for near maximum doses [3] and the GEC/ESTRO recommendation is to limit the D_2cc_ to 75 Gy [4]. Additionally, the identified seriality is consistent with the suggestion rectal bleeding is associated with epithelial damage and mucositis as a result of exposure of parts of the rectal wall to near maximum doses [31].

### The mid-dose region is important for bleeding/non-bleeding toxicities

Studies have also demonstrated that the mid-dose region (>30 Gy) is important for rectal bleeding [28, 30, 32, 33]. In this study the importance is shifted to relatively higher doses in the mid-high dose range for both distribution-adding and parameter-adding. An influencing factor for the lack of importance of the lower end of the mid-dose range could be that the combined EBRT/HDR treatments were subject to the constraint that the maximum rectum dose from HDR should not exceed 80 % of the 19.5 Gy prescription dose for HDR. Consequently, in the context of the total EBRT/HDR dose this constraint effectively applies more to the lower end of the mid-dose range after adjusting for dose fractionation than it does to the high-dose region. The importance of the high-dose and near maximum dose regions could also be related to the steepness of dose gradients associated with HDR treatments as it has been proposed that a focused high-dose region could aid healing of the vascular sclerosis in high-dose regions via cell migration from the low-dose region [50]. Consequently, it would be useful to determine optimal rectum dose constraints for combined EBRT/HDR based on accumulated dose. A larger sample size containing patients from a variety of institutions would allow for a feasible application of multivariate and cut-point analysis.

The upper end of the mid-high dose range after distribution-adding was important for the non-bleeding toxicities of stool frequency and urgency/tenesmus. This result could support the earlier suggestion that the rectum dose constraint for HDR effectively applies a constraint to the lower end of the mid-dose range when the total EBRT/HDR dose is considered. The dose constraint in one dose region leading to other dose regions becoming important is consistent with a previous study focusing on patients within this trial who received EBRT only [30]. The study indicated the low-mid dose range was important for stool frequency, urgency and tenesmus in the presence of high-dose constraints [30]. More optimised dose constraints for the mid-high dose range based on accumulated dose could be useful for reducing the toxicities associated with these doses. Such constraints could be relatively more important for urgency/tenesmus compared to stool frequency given the higher toxicity rate in this patient sample compared to the other toxicities.

### Toxicity is also influenced by low doses and the lower end of the mid-dose range

The association of urgency/tenesmus with distribution-adding doses at the lower end of the mid-dose range is consistent with the finding from another study where violation of the V_40 Gy_ dose constraint was important for urgency [26]. Additionally, the results indicate the lower end of the mid-dose range and the low doses may be associated with anorectal pain and rectal bleeding respectively. The correlation of toxicities with low doses and the lower end of the mid-dose range is possible as it is plausible that a low-dose bath to a large volume will be associated with detriment. However, these findings of association should be considered with respect to toxicity event rates, sample size and the potential of random discovery.

### Software developments to improve contour consistency and registration accuracy for the prostate/rectum interface would be of great benefit

Analysis based on contouring and registration is associated with uncertainties. However, the dose regions indicated as being important for toxicity after distribution-adding were in most cases consistent with those indicated as important after parameter-adding. The low-mid dose range for parameter-adding was significantly associated with proctitis and stool frequency. In contrast, these regions after distribution-adding were not identified as important. However, distribution-adding did indicate additional regions as important compared to regions identified by the parameter-adding results. For example, the analysis for parameter-adding did not indicate any significant dose regions for diarrhoea and urgency/tenesmus whereas analysis after distribution-adding indicated the mid-high dose range was important. The alpha-beta ratio is an additional uncertainty for diarrhoea correlations as the mid-high dose range was only important for an alpha-beta ratio of 3 Gy. Further studies for a variety of contouring and registration contexts would be useful for gathering data for the purpose of determining whether registration and distribution-adding reveals correlations which were not identified by parameter-adding.

When considering the distribution-adding findings in isolation it should be noted that errors in contouring and registration accuracy will confound the distribution-adding parameters that have been correlated with toxicity. A median overlap of 80.4 % for the rectum volume correspondence across all patients would indicate the registrations are satisfactory as a general structure overlap of 70 % is considered to be the starting point for satisfactory structure-correspondence in the radiotherapy context [22, 23]. The proximity of the HDR rectum to the HDR catheters makes parameters obtained by registration and distribution-adding sensitive to small localised variations in contouring and/or registration accuracy across the prostate/rectum interface. Given this is the first study to accumulate the rectum dose for combined EBRT/HDR prostate cancer treatments using deformable registration and then correlate the doses with toxicity, we encourage more prostate cancer studies to assess the importance of dose-volume metrics using a variety of registration algorithms. Software developers and treatment planning vendors have the opportunity to greatly improve planning and the reliability of dose-toxicity modelling after registration by improving contouring and registration accuracy for the prostate/rectum interface.

### Inter-fraction motion should be considered

A common uncertainty associated with EBRT, HDR and other radiotherapy techniques is inter-fraction motion of patient anatomy [51–54]. In response to this uncertainty, it is becoming more common for institutions to adopt repeat imaging over the course of prostate cancer treatment to correct for inter-fraction motions and improve the correspondence between planned dose and delivered dose [53, 55]. However, many studies including this study do not contain repeat imaging due to the retrospective nature of studies where long follow-up is required to correlate dose with late toxicities. Consequently, studies are constrained by treatments performed in the past with the associated resources and protocols at that time.

A consideration for prostate EBRT in this study is rectum motion and variable rectum contents confounding the accuracy of rectum dose distributions obtained from single static planning CTs [52, 56, 57]. A variety of methods have been used to estimate the impact of inter-fraction motion on rectum dose parameters and dose-response modelling for prostate EBRT [8, 52, 58–61]. To obtain appropriate mean estimates for the difference in EBRT rectum dose between the single CT based values and the motion-corrected values this study analysed the results of another study [52] that used the same registration software and registration algorithm. Consequently, compared to the motion-corrected values the single CT based values may be conservative estimates by 3.9 % for the D_2%_ and 5.8 % for the equivalent uniform dose [52].

An important consideration for inter-fraction motion during prostate brachytherapy is the movement of the anterior rectal wall relative to the prostate [54, 62]. A variety of methods have been used to estimate inter-fraction motion in prostate HDR [16, 54] and the subsequent impact on rectum dose parameters for prostate brachytherapy [54, 63–65]. Simnor et at. [54] calculated that the catheter mean caudal displacements of 7.9 mm and 3.8 mm prior to fractions 2 and 3 were associated with mostly systematic increases to the D_2cc_ of 0.69 Gy (~ 6.6 %) and 0.76 Gy (~ 7.2 %) respectively. For HDR at the institution where patients in this study were treated, the displacement of catheters was checked prior to each of the three fractions using an anterior-posterior radiograph and corrected for using a rigid external holding device as described by Tiong et al. [16]. The catheter mean caudal displacements after this advancement process were reported as 1.7 mm, 1.1 mm and 0.8 mm for fractions 1, 2 and 3 respectively [16]. Consequently, the inter-fraction motion increases to the D_2cc_ reported by Simnor et al. [54] may be appropriate conservative estimates for the inter-fraction motion of HDR catheters that could be expected for this study.

It is possible that the above mentioned inter-fraction motion could remove significance of dose ranges. However, shifting of dose values identified as significant are likely as the single CT based estimates were mostly identified as being systematically different to the motion-corrected values [52, 54]. The influence of inter-fraction on delivered doses is likely to be important when considering dose constraints recommended by studies where planned dose tends to be less than delivered dose. Consequently, it would be useful to confirm the importance of published dose-volume constraints after registration is applied for repeat daily imaging.

### Avenues and recommendations for further analysis

- Given this is the first study to apply deformable registration prior to correlating combined EBRT/HDR dose with toxicity, it is important that the model and findings be validated in other contexts with standardised contouring, implanting and planning guidelines for EBRT and HDR.
- A larger sample size would make it feasible to explore models that incorporate multiple toxicity events over the follow-up period [66] or include the persistence of toxicity rather than peak late toxicity [67].
- Image guided radiotherapy or further imaging could improve the reliability of accumulated dose-histogram metrics [13].
- Customised registration algorithms for accurately handling the catheters within the HDR prostate or data for treatments which use plastic HDR catheters are encouraged as prostate and urethra doses are key clinical concerns in the RADAR trial [27].
- It would also be useful to determine whether other aspects of the total registered dose distribution add predictive capability to dose-toxicity modelling e.g. including dose-shape toxicity modelling [60].
- Exploring the association between toxicity and doses to other organs or regions may be useful for further explaining the incidence of toxicity (e.g. doses to the bowel and gastrointestinal tract could be associated with toxicity [46, 68]).

## Conclusions

A number of significant dose-histogram effects were revealed for gastrointestinal toxicities after applying deformable registration to adjust for the anatomical differences between planning CTs for each phase of a combine EBRT/HDR prostate cancer treatment. The findings for distribution-adding were in most cases consistent with those for parameter-adding. The mid-high dose range and near maximum doses were important for rectal bleeding. The distribution-adding mid-high dose range was also important for stool frequency and urgency/tenesmus. The anorectum doses which were important for toxicity are reported to guide and encourage future planning of combined EBRT/HDR prostate cancer treatments based on accumulated phases with appropriate inter-fraction motion management. We encourage other studies to report on important dose-histogram effects and spatial aspects of accumulated dose distributions for combined EBRT/HDR.

## Additional file

Additional file 1: Online supplementary material (Supplements A-H.pdf) providing additional treatment details, patient characteristics, visual checks of registrations, results which were not significant, results for α/β = 5.4 Gy and results for parameter-adding. (PDF 2267 kb)

## Abbreviations

CERR: Computational environment for radiotherapy research
CI: Confidence intervals
CTCAE: Common toxicity criteria for adverse events
DIR: Deformable image registration
DVH: Dose-volume histograms
D_X%_: Minimum dose to the most irradiated X percent of the rectal volume after applying an alpha-beta ratio
EBRT: External beam radiotherapy
EQDX_α/β_ Gy: Equieffective dose given in a reference X Gy per fraction using α/β
HDR: High-dose-rate brachytherapy
LDR: Low-dose-rate brachytherapy
LENT SOMA: Late effects of normal tissue — subjective, objective, management and analytic
OR: Odds ratio
RADAR: Randomized androgen deprivation and radiotherapy trial
TROG: Trans-Tasman Radiation Oncology Group
V_X_: Percentage of the rectal volume receiving at least X Gy after applying an alpha-beta ratio
α/β: Alpha-beta ratio
