# Supplementary material for: Prostate external beam radiotherapy combined with high-dose-rate brachytherapy: dose-volume parameters from deformably-registered plans correlate with late gastrointestinal complications
Source: Radiat Oncol. 2016 Oct 31;11:144. doi: 10.1186/s13014-016-0719-2 (PMC5087115; doi:10.1186/s13014-016-0719-2)
Supplement: Supplementary file 1 — Online supplementary material (Supplements A-H.pdf) providing additional treatment details, patient characteristics, visual checks of registrations, results which were not significant, results for α/β = 5.4 Gy and results for parameter-adding. (PDF 2267 kb) [file 13014_2016_719_MOESM1_ESM.pdf]

Supplementary Material

Calyn R. Moulton[1], Michael J. House[1], Victoria Lye[2], Colin I. Tang[2], Michele Krawiec[2], David J. Joseph[2,3], James W. Denham[4] and Martin A. Ebert[1,2]

[1]School of Physics, University of Western Australia, Crawley, Western Australia, Australia;
[2]Radiation Oncology, Sir Charles Gairdner Hospital, Nedlands, Western Australia, Australia;
[3]School of Surgery, University of Western Australia, Crawley, Western Australia, Australia;
[4]School of Medicine and Population Health, University of Newcastle, Callaghan, New South Wales, Australia.

List of sections:

Supplement A. Patient characteristics and treatment protocol

Supplement B. Examples of planning CTs and planned doses

Supplement C. System for grading toxicity

Supplement D. Assessing registration misalignment

Supplement E. Distribution-adding results which were not significant for other end points with α/β=3 Gy

Supplement F. Parameter-adding results for α/β=3 Gy

Supplement G. Distribution-adding results for α/β=5.4 Gy

Supplement H. Parameter-adding results for α/β=5.4 Gy

Supplement A. Patient characteristics and treatment protocol

The 118 prostate cancer patients were initially treated with neoadjuvant androgen deprivation therapy at Sir Charles Gairdner Hospital in the period 2004 to 2008. Then they received EBRT followed by HDR. Patient criteria and treatment methodology were as specified for the Trans-Tasman Radiation Oncology Group (TROG) 03.04 Randomized Androgen Deprivation and Radiotherapy (RADAR) trial [1, 2]. The standard HDR planning and treatment process has previously been described [3]. The important details are:

- The EBRT prescription dose to the prostate for the 118 patients was 46 Gy to the International Commission on Radiation Units and Measurements 50 reference point (23 daily fractions of conventional fractionation over 5 weeks).
- The four-field three-dimensional EBRT plan was created in the Elekta XiO treatment planning system (Elekta AB, Stockholm, Sweden) based on the planning CT with the patient in the supine position.
- The prescription dose to the prostate was 19.5 Gy for HDR delivered by Iridium-192 after-loading catheters (Varian Oncology Systems).
- The HDR prescription dose covered the prostate gland and any extracapsular extensions.
- The HDR was delivered in 3 fractions of 6.5 Gy across 2 days with a maximum delivery time of 90 minutes for each fraction and a minimum of 6 hours between fractions.
- The dose to the rectum from HDR was limited to a maximum of 80% of the 19.5 Gy prescription dose.
- The HDR was typically started 2 to 5 weeks after the end of external beam radiotherapy.
- The temporary metal needle HDR catheters were inserted with trans-rectal ultrasound, fluoroscopy and perineal template guidance while the patient was in the lithotomy position.
- Needle catheters were inserted and cystoscopy was used to ensure proper tenting of the bladder wall mucosa by catheters.
- A plastic template was sutured to the skin to hold the needles in place.
- The patient was then taken to have a HDR planning CT and a 3 fraction HDR plan was created in the BrachyVision treatment planning system (Varian Medical Systems, Palo Alto, US) based on this CT.
- The HDR doses were based on the standard TG43 format [4].
- Patients were in the lithotomy position for HDR treatment with cushions used to keep the patients' legs in the abducted position between fractions.
- No patient had artificial hip joints.
- The EBRT planning target volume (PTV) and HDR source definition volume (SDV) were obtained by expanding the corresponding clinical target volume (CTV) by a 10 mm margin. The HDR SDV was used to restrict dwell positions to the lengths of identified catheters covered by the SDV.

Table A1 summarises the patient characteristics at baseline.

Table A1. The baseline clinical characteristics of 118 patients who underwent external beam radiotherapy followed by high-dose-rate brachytherapy for prostate cancer.

Characteristic	Aspect of characteristic	Value
Age	Median	66.6
	Interquartile range	61.0-71.4
PSA	PSA < 10	31 (26.3%)
	10 ≤ PSA < 20	42 (35.6%)
	PSA ≥ 20	45 (38.1%)
Gleason score	< 7	2 (1.7%)
	= 7	41 (34.7%)
	≥ 8	75 (63.6%)
Tumour classification	T2b	14 (11.9%)
	T2c	16 (13.6%)
	T3	87 (73.7%)
	T4	1 (0.8%)
Risk group	Medium	29 (24.6%)
	High	89 (75.4%)
Number of HDR catheters	12	2 (1.7%)
	13	3 (2.5%)
	14	2 (1.7%)
	15	96 (81.4%)
	16	2 (1.7%)
	17	13 (11.0%)

Abbreviations: PSA = Prostate-specific antigen; HDR = High-dose-rate brachytherapy

Supplement B. Examples of planning CTs and planned doses

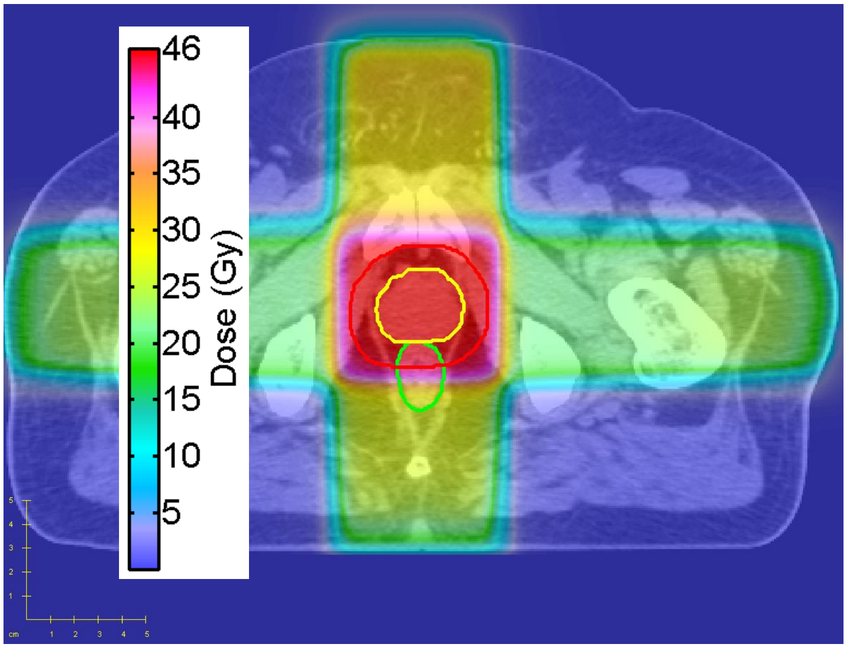

Fig. A1. A four-field EBRT physical dose plan with dose displayed as a colourwash up to the prescription dose of 46 Gy. The EBRT clinical target volume, planning target volume and rectum structures are in yellow, red and green respectively.

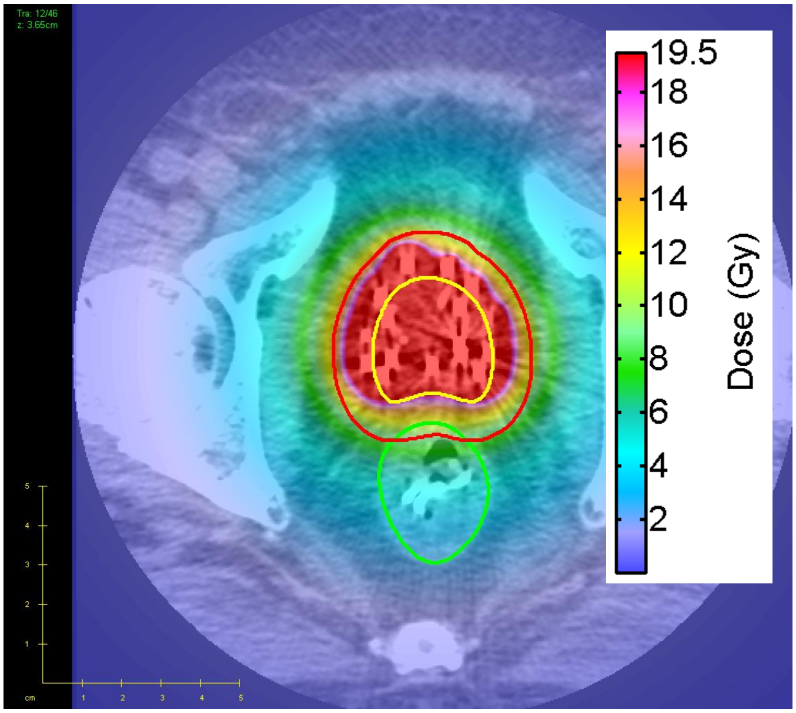

Fig. A2. A HDR TG43 physical dose plan with dose displayed as a colourwash up to the prescription dose of 19.5 Gy. The HDR clinical target volume, source definition volume and rectum structures are in yellow, red and green respectively.

Supplement C. System for grading toxicity

Table A2. Toxicity grading system for clinician assessed rectal bleeding, stool frequency, diarrhoea, completeness of evacuation, anorectal pain, urgency and tenesmus, and CTC proctitis.

Toxicity	Grade 0	Grade 1	Grade 2	Grade 3	Grade 4
Rectal bleeding	Never	Occult	> 2/week	Daily	Gross haemorrhaging
Stool frequency	< 2/day	2-4/day	5-8/day	> 8/day	Uncontrolled diarrhoea
Diarrhoea	None	Increase < 4 stools/day	Increase of 4-6 stools/day or nocturnal stools	Increase of ≥ 7 stools/day or incontinence or parenteral support	Physiologic consequences requiring intensive care; haemodynamic collapse
Completeness of evacuation	Complete evacuation (“requires one movement to completely empty bowel or feel you’re all done”)	Occasional multiple evacuations (“about once a week feel like you’re not ‘all done’ or it takes more than one movement to finish”)	Frequent multiple evacuations (“more than once a week feel like you’re not ‘all done’ or it takes more than one movement to finish”)	Requires enema to obtain complete emptying	--
Anorectal pain	Never	Occasional and mild	Intermittent and tolerable	Persistent and intense	Refractory and excruciating
Urgency and tenesmus	Never	Occasional	Intermittent	Persistent	Refractory
CTC proctitis	None	Increased stool frequency, occasional blood-streaked stools or rectal discomfort not requiring medication	Increased stool frequency, bleeding mucous discharge or rectal discomfort requiring medication, anal fissure	Increased stool frequency/diarrhoea requiring parenteral support, rectal bleeding requiring transfusion, or persistent mucous discharge necessitating pads	Perforation, bleeding or necrosis or other life threatening complication requiring surgical intervention

Supplement D. Assessing registration misalignment

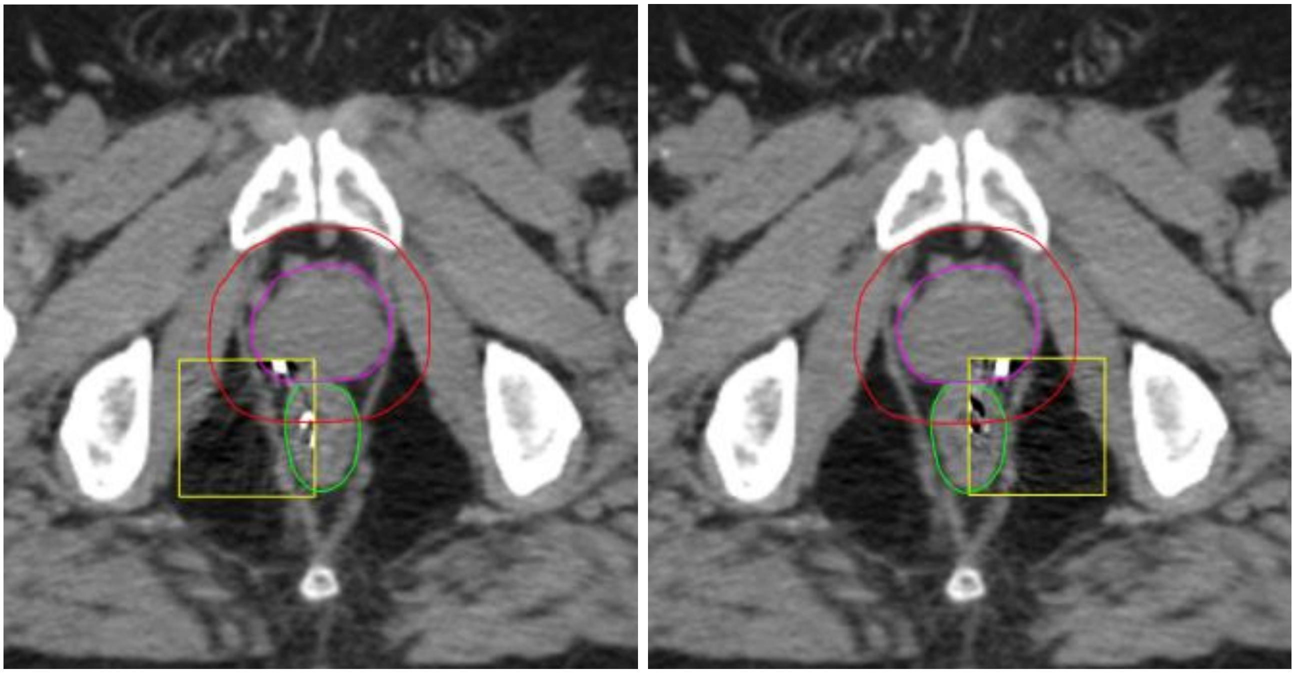

Fig. A3. Use of the spyglass box tool in Velocity Advanced Imaging to check the anatomical alignment between images. The EBRT images are in the background. Regions of the HDR images after a rigid plus multi-pass deformable image registration are contained within the yellow outlined rectangular spyglass box. This box can be resized and moved around (e.g. left image versus right image). The EBRT clinical target volume, planning target volume and rectum structures are in purple, red and green respectively.

Supplement E. Distribution-adding results which were not significant for other end points with α/β=3 Gy

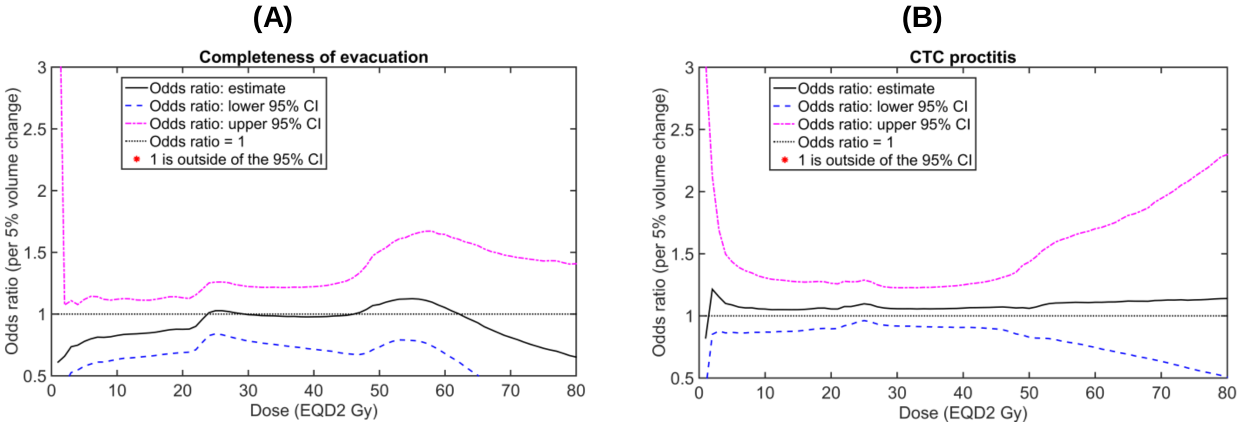

Fig. A4. Odds ratios from univariate ordinal regression of distribution-adding V_X and peak late toxicity for completeness of evacuation **(A)** and proctitis **(B)**. The peak late toxicities for completeness of evacuation were dichotomised at grade 2 where as proctitis was dichotomised at grade 1. A red dot is used to indicate the doses at which odds ratios are significantly different from a value of one (95% confidence intervals do not include one). *Abbreviations:* V_X, percentage of the rectal volume receiving at least X Gy after applying an α/β=3 Gy; EQD2 Gy, equivalent dose in 2-Gy fractions using α/β=3 Gy; 95% CI, 95% confidence interval.

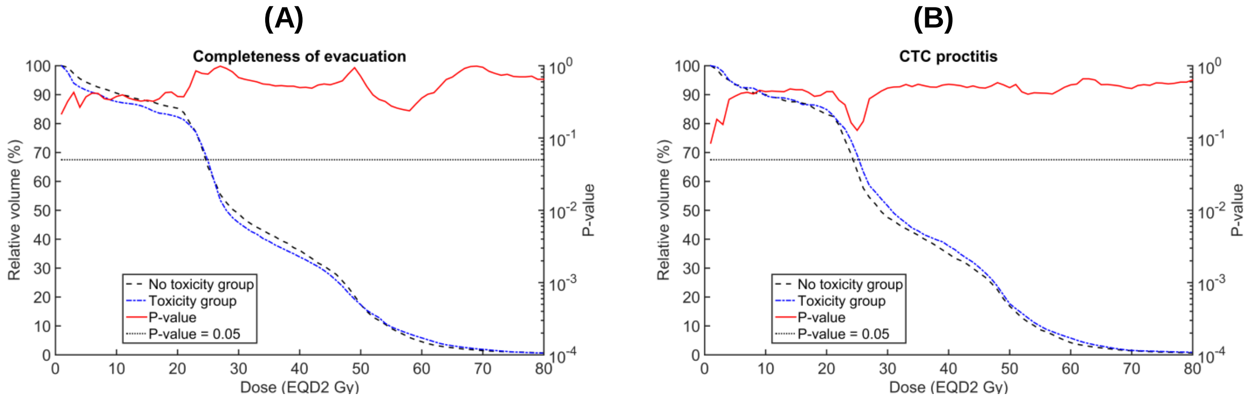

Fig. A5. Median distribution-adding V_X for the toxicity and no toxicity groups. The groups are based on peak late toxicity for completeness of evacuation **(A)** and proctitis **(B)**. The peak late toxicities for completeness of evacuation were dichotomised at grade 2 where as proctitis was dichotomised at grade 1. The red curve and p-value axis indicate doses at which median V_X values for the toxicity and no toxicity groups are significantly different ($p\text{-value} < 0.05$). *Abbreviations:* V_X, percentage of the rectal volume receiving at least X Gy after applying an $\alpha/\beta=3$ Gy; EQD2 Gy, equivalent dose in 2-Gy fractions using $\alpha/\beta=3$ Gy.

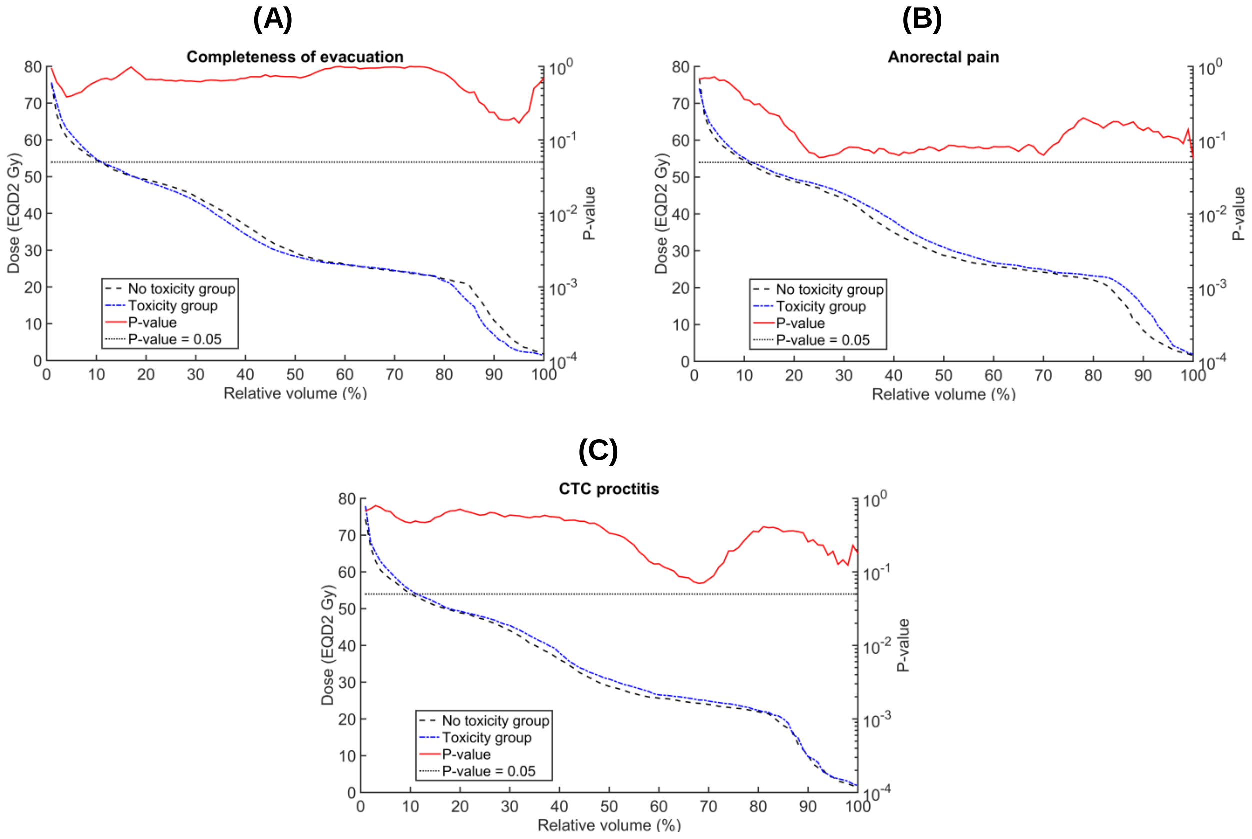

Fig. A6. Median distribution-adding $D_{X\%}$ for the toxicity and no toxicity groups. The groups are based on peak late toxicity for completeness of evacuation **(A)**, anorectal pain **(B)** and proctitis **(C)**. The peak late toxicities for completeness of evacuation were dichotomised at grade 2 whereas anorectal pain and proctitis were dichotomised at grade 1. The red curve and p-value axis indicate doses at which median $D_{X\%}$ values for the toxicity and no toxicity groups are significantly different (p-value < 0.05). *Abbreviations:* $D_{X\%}$, minimum dose to the most irradiated X percentage of rectal volume after applying an α/β=3 Gy; EQD2 Gy, equivalent dose in 2-Gy fractions using α/β=3 Gy.

Supplement F. Parameter-adding results for α/β=3 Gy

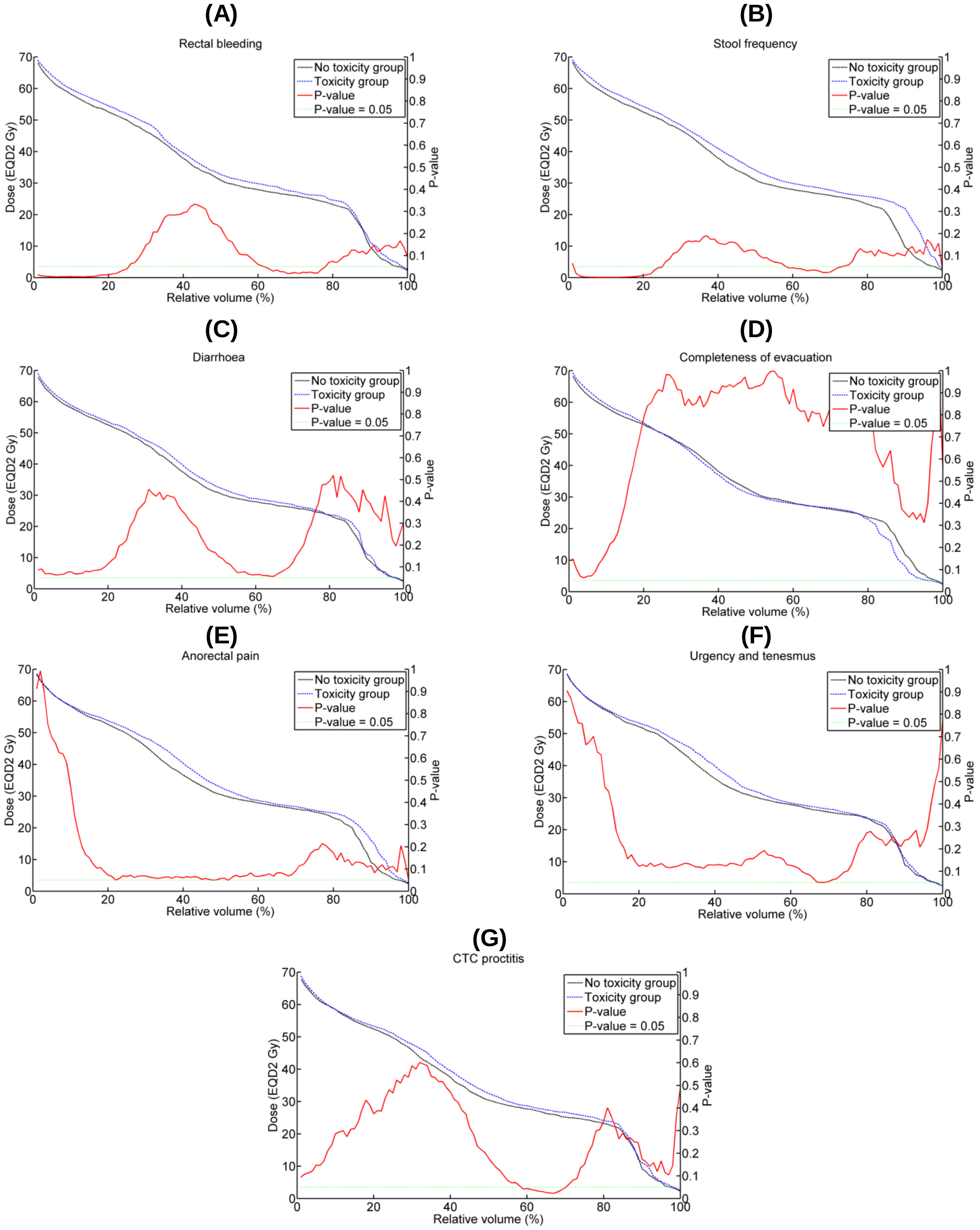

Fig. A7. Median parameter-adding $D_{X\%}$ for the toxicity and no toxicity groups. The peak late toxicities for rectal bleeding **(A)**, stool frequency **(B)** and completeness of evacuation **(D)** were dichotomised at grade 2 whereas diarrhoea **(C)**, anorectal pain **(E)**, proctitis **(G)** and urgency/tenesmus **(F)** were dichotomised at grade 1. The red curve and p-value axis indicate doses at which median $D_{X\%}$ values for the toxicity and no toxicity groups are significantly different (p-value < 0.05). *Abbreviations:* $D_{X\%}$, minimum dose to the most irradiated X percentage of rectal volume after applying parameter-adding and an α/β=3 Gy; EQD2 Gy, equivalent dose in 2-Gy fractions using α/β=3 Gy.

Supplement G. Distribution-adding results for α/β=5.4 Gy

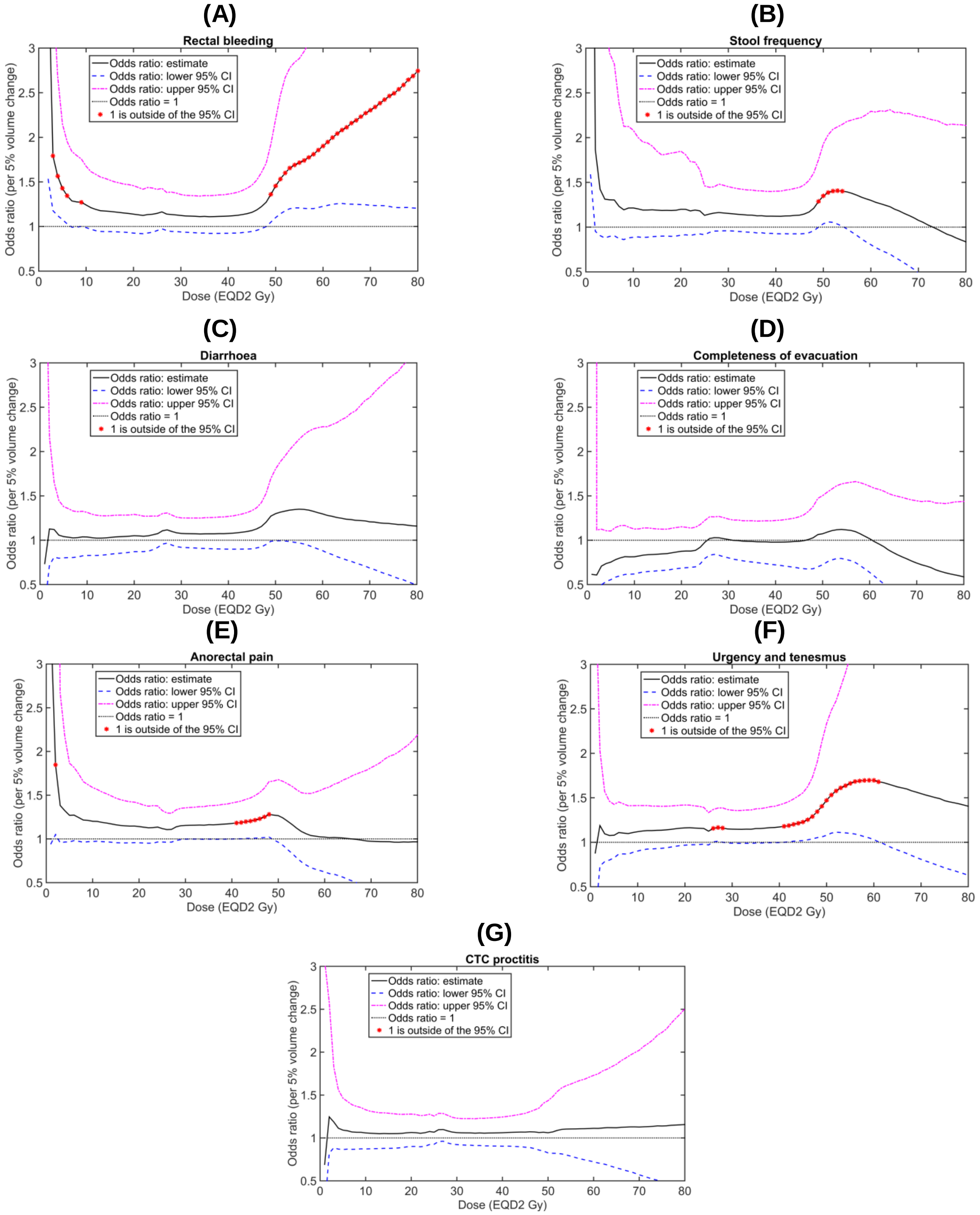

Fig. A8. Odds ratios from univariate ordinal regression of distribution-adding V_X and peak late toxicity for rectal bleeding **(A)**, stool frequency **(B)**, diarrhoea **(C)**, completeness of evacuation **(D)**, anorectal pain **(E)**, urgency/tenesmus **(F)** and proctitis **(G)**. The peak late toxicities for rectal bleeding, stool frequency and completeness of evacuation were dichotomised at grade 2 whereas diarrhoea, anorectal pain, proctitis and urgency/tenesmus were dichotomised at grade 1. A red dot is used to indicate the doses at which odds ratios are significantly different from a value of one (95% confidence intervals do not include one). *Abbreviations:* V_X, percentage of the rectal volume receiving at least X Gy after applying an α/β=5.4 Gy; EQD2 Gy, equivalent dose in 2-Gy fractions using α/β=5.4 Gy; 95% CI, 95% confidence interval.

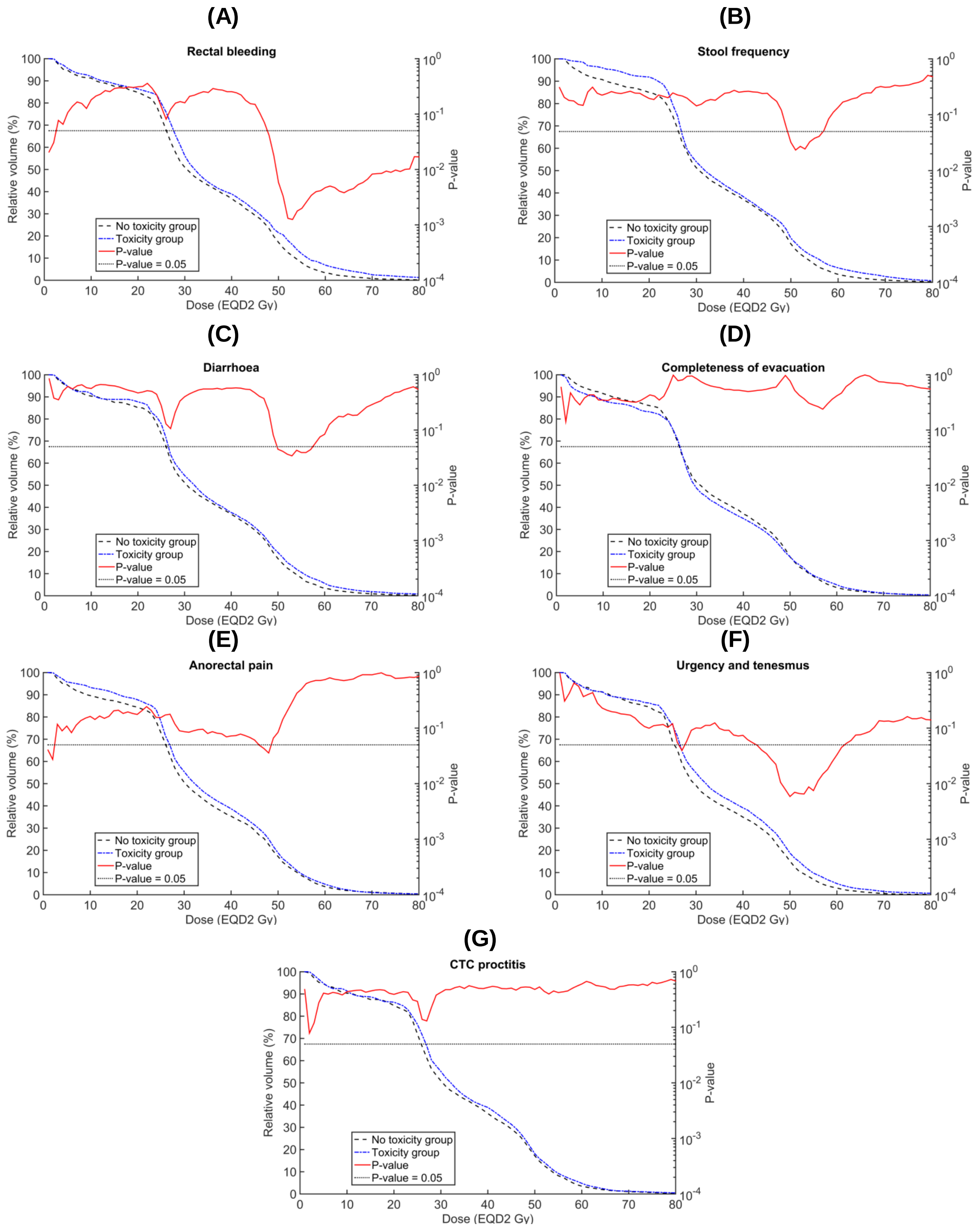

Fig. A9. Median distribution-adding V_X for the toxicity and no toxicity groups. The groups are based on peak late toxicity for rectal bleeding **(A)**, stool frequency **(B)**, diarrhoea **(C)**, completeness of evacuation **(D)**, anorectal pain **(E)**, urgency/tenesmus **(F)** and proctitis **(G)**. The peak late toxicities for rectal bleeding, stool frequency and completeness of evacuation were dichotomised at grade 2 whereas diarrhoea, anorectal pain, proctitis and urgency/tenesmus were dichotomised at grade 1. The red curve and p-value axis indicate doses at which median V_X values for the toxicity and no toxicity groups are significantly different (p-value < 0.05). *Abbreviations:* V_X, percentage of rectal volume receiving at least X Gy after applying an α/β=5.4 Gy; EQD2 Gy, equivalent dose in 2-Gy fractions using α/β=5.4 Gy.

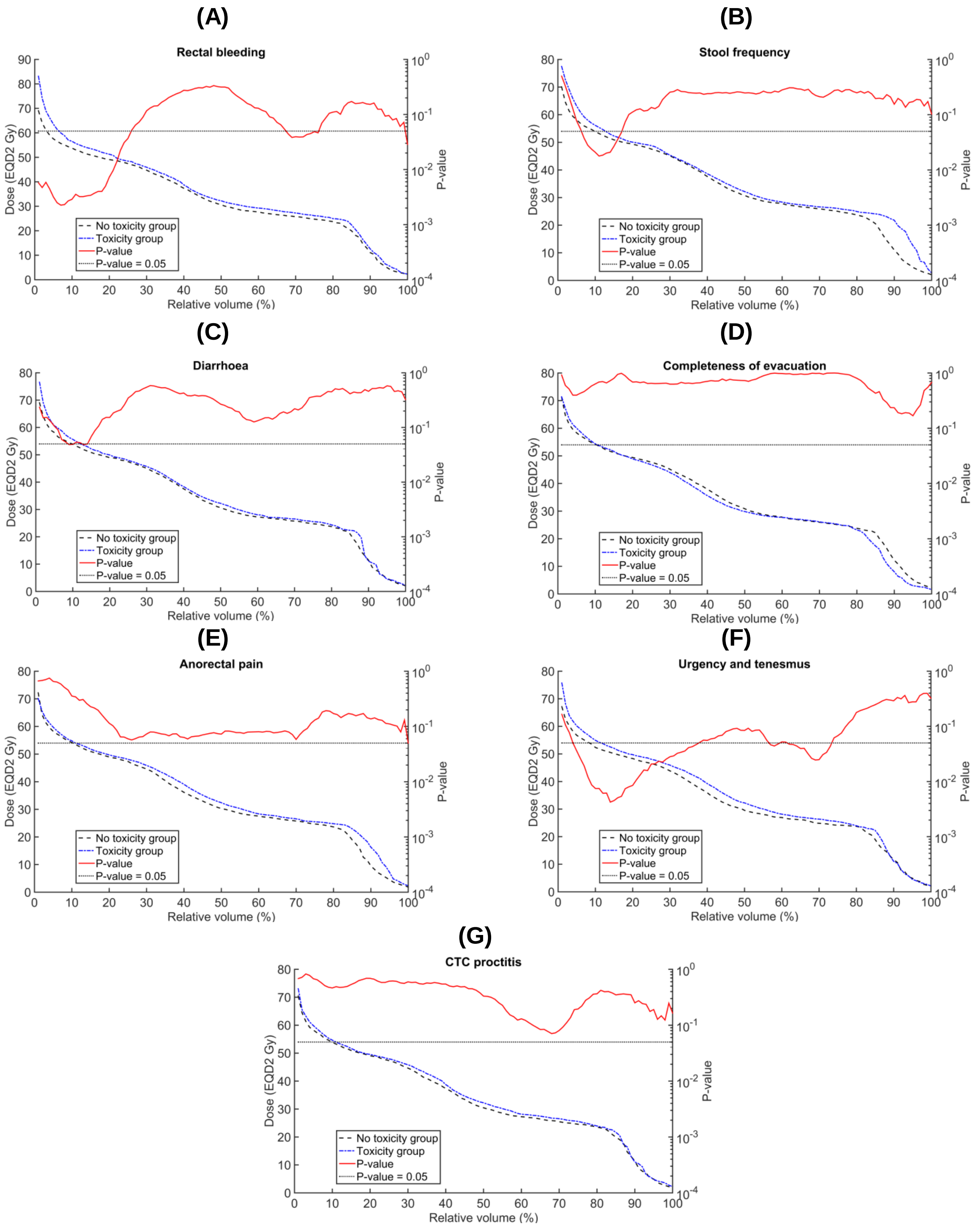

Fig. A10. Median distribution-adding $D_{X\%}$ for the toxicity and no toxicity groups. The groups are based on peak late toxicity for rectal bleeding **(A)**, stool frequency **(B)**, diarrhoea **(C)**, completeness of evacuation **(D)**, anorectal pain **(E)**, urgency/tenesmus **(F)** and proctitis **(G)**. The peak late toxicities for rectal bleeding, stool frequency and completeness of evacuation were dichotomised at grade 2 whereas diarrhoea, anorectal pain, proctitis and urgency/tenesmus were dichotomised at grade 1. The red curve and p-value axis indicate doses at which median $D_{X\%}$ values for the toxicity and no toxicity groups are significantly different (p-value < 0.05). *Abbreviations:* $D_{X\%}$, minimum dose to the most irradiated X percentage of rectal volume after applying an α/β=5.4 Gy; EQD2 Gy, equivalent dose in 2-Gy fractions using α/β=5.4 Gy.

Supplement H. Parameter-adding results for α/β=5.4 Gy

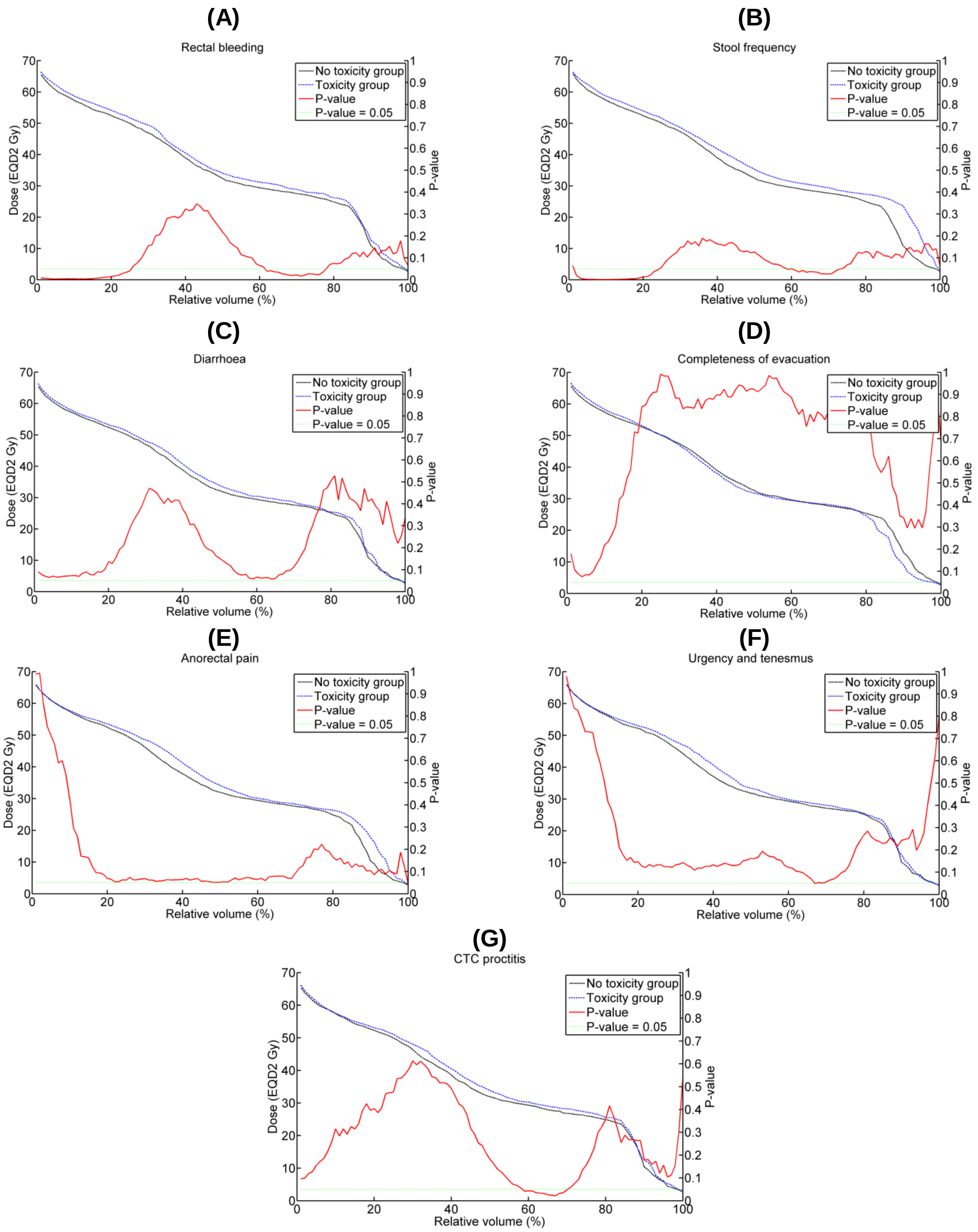

Fig. A11. Median parameter-adding $D_{X\%}$ for the toxicity and no toxicity groups. The peak late toxicities for rectal bleeding **(A)**, stool frequency **(B)** and completeness of evacuation **(D)** were dichotomised at grade 2 whereas diarrhoea **(C)**, anorectal pain **(E)**, proctitis **(G)** and urgency/tenesmus **(F)** were dichotomised at grade 1. The red curve and p-value axis indicate doses at which median $D_{X\%}$ values for the toxicity and no toxicity groups are significantly different (p-value < 0.05). *Abbreviations:* $D_{X\%}$, minimum dose to the most irradiated X percentage of rectal volume after applying parameter-adding and an α/β=5.4 Gy; EQD2 Gy, equivalent dose in 2-Gy fractions using α/β=5.4 Gy.

References

1. Denham JW, Joseph D, Lamb DS, Spry NA, Duchesne G, Matthews J et al. Short-term androgen suppression and radiotherapy versus intermediate-term androgen suppression and radiotherapy, with or without zoledronic acid, in men with locally advanced prostate cancer (TROG 03.04 RADAR): an open-label, randomised, phase 3 factorial trial. Lancet Oncol. 2014; doi:10.1016/S1470-2045(14)70328-6.
2. Denham JW, Steigler A, Joseph D, Lamb DS, Spry NA, Duchesne G et al. Radiation dose escalation or longer androgen suppression for locally advanced prostate cancer? Data from the TROG 03.04 RADAR trial. Radiother Oncol. 2015; doi:10.1016/j.radonc.2015.05.016.
3. Tiong A, Bydder S, Ebert M, Caswell N, Waterhouse D, Spry N et al. A small tolerance for catheter displacement in high-dose rate prostate brachytherapy is necessary and feasible. Int J Radiat Oncol Biol Phys. 2010; doi:10.1016/j.ijrobp.2009.03.052.
4. Rivard MJ, Coursey BM, DeWerd LA, Hanson WF, Huq MS, Ibbott GS et al. Update of AAPM Task Group No. 43 Report: A revised AAPM protocol for brachytherapy dose calculations. Med Phys. 2004; doi:10.1118/1.1646040.
